# Robust DUT-67 material for highly efficient removal of the Cr(VI) ion from an aqueous solution

**DOI:** 10.3389/fchem.2023.1148073

**Published:** 2023-02-28

**Authors:** Yanqiong Shen, Qingsong Yang, Yongqiang Gao, Jinjie Qian, Qipeng Li

**Affiliations:** ^1^ College of Chemistry and Chemical Engineering, Zhaotong University, Zhaotong, China; ^2^ Shuifu No 1 Middle School, Zhaotong, China; ^3^ Kunming Real-E Foreign Language Middle School, Kunming, China; ^4^ College of Chemistry and Materials Engineering, Wenzhou University, Wenzhou, China

**Keywords:** DUT-67, highly efficient, removal of the Cr(VI) ion, aqueous solution, highly stable

## Abstract

Robust **DUT-67** was synthesized by the hydrothermal method and characterized by powder X-ray diffraction (PXRD), scanning electron microscopy (SEM), and thermogravimetric analysis (TGA). To systematically study the removal of Cr(VI) ion by **DUT-67**, single-factor, competition ion, material regeneration, kinetic, and thermodynamic experiments were designed. The experimental results show that **DUT-67** had a maximum removal rate of 96.1% and a maximum adsorption capacity of 105.42 mg g^−1^ with material regeneration and outstanding selective adsorption. In addition, the process of removal of the Cr(VI) ion from an aqueous solution by **DUT-67**, which accorded with the pseudo-second-order kinetics model and Langmuir model, was studied, and its adsorption mechanism was reasonably explained by the theoretical calculation.

## 1 Introduction

Chromium pollution due to the highly toxic Cr(VI) ion, which is easily absorbed by the human body through a variety of pathways, causes long-term harm to the environment and poses a great threat to human health, causing damage to the body, hoarse throats, perforation of the nasal septum, emphysema, lung sclerosis, and other diseases (Liu et al., 2020; Bell et al., 2022; Shen et al., 2022). Therefore, strict checks on the emission standards of Cr(VI) are necessary, requiring effective treatment of industrial waste with Cr(VI) ions (Wang et al., 2013). So far, the removal methods of the Cr(VI) ion mainly include flotation, precipitation, reverse osmosis, vaporization, bio-treatment and chemical oxidation, adsorption, ion exchange, and a combination of these methods (Gao et al., 2019; Li et al., 2021). The adsorption method was considered the most direct, simple, and effective method to treat the industrial waste with the Cr(VI) ion, both in industry and research (Schoenecker et al., 2012; Wang et al., 2016).

In recent years, porous materials such as large-pore resin, activated carbon, metal–organic frameworks (MOFs), natural zeolite, silica gel, molecular sieve, and covalent organic frameworks have significantly advanced in the field of adsorption and they interact favorably during separation and purification (Peng, et al., 2018; Wychowaniec et al., 2022). Metal–organic frameworks have high porosity, large surface area, high stability, and simple preparation when compared with the traditional porous materials (Bai et al., 2016; Zhang et al., 2021). In addition, the Cr(VI) ion in the nano-pore or nano-cage of MOFs can interact with active sites, facilitating the efficient removal of the Cr(VI) ion. So far, only few MOFs have high stability, such as ZIFs, MILs, and UiO-X (Wang et al., 2016; Zhang et al., 2021), which have been applied in the removal of the Cr(VI) ion. Although some progress on the highly stable porous MOF materials for the removal of the Cr(VI) ion has been achieved (Li et al., 2013; Zhang et al., 2015; Shen et al., 2022), the design of highly stable MOFs and application in the efficient removal of the Cr(VI) ion from wastewater remain a challenging work.

In this work, robust **DUT-67** was used for the removal of the Cr(VI) ion from an aqueous solution, and the single-factor, competition ion, material regeneration, kinetic, and thermodynamic experiments were performed. In addition, the removal process by **DUT-67** was analyzed *via* dynamic and thermodynamic modeling. The removal process of the Cr(VI) ion by **DUT-67** was explored, and the adsorption mechanism was speculated through theoretical calculation.

## 2 Results and discussion

### 2.1 Structural and morphological characterization

**FIGURE 1:**
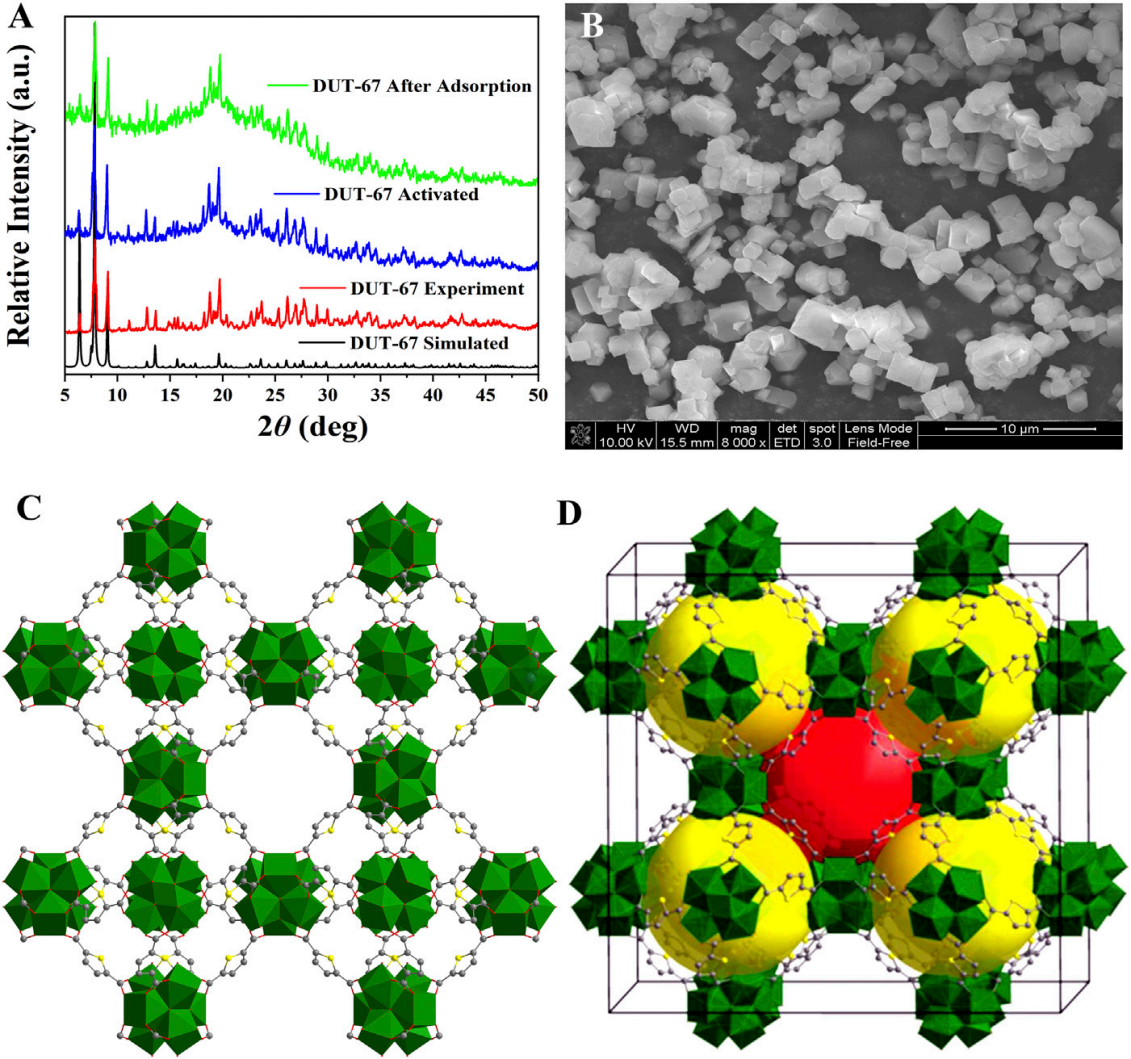
XRD **(A)**, SEM **(B)**, and the three-dimensional structure **(C, D)** of **DUT-67**.

2,5-Thiophenedicarboxylic acid (H_2_TDC) and ZrCl_4_ were used to prepare **DUT-67** using the hydrothermal method, and its structure, morphology, and thermal stability were characterized by PXRD, SEM, and TGA, respectively. The PXRD results show that the characteristic peaks of the experimental and simulated **DUT-67** are consistent, indicating the successful preparation of **DUT-67**. In addition, the characteristic peak positions of the activated and reused samples of **DUT-67** are unchanged, indicating the high-temperature activated and reused samples of **DUT-67** still maintain the crystal state (Figure 1A). The morphology of **DUT-67** is observed as a white powder with regular morphology but uneven size (Figure 1B and Supplementary Figure S1). The TGA results showed that **DUT-67** lost the guest solvent molecules between 25°C and 300°C, while the frameworks began to decompose after 350°C, indicating that **DUT-67** exhibits high thermal stability (Supplementary Figure S2). **DUT-67** can exist stably in concentrated HCl for 3 days, which indicates the chemical stability (Bon et al., 2013) of **DUT-67**. Therefore, **DUT-67** exhibits both high thermal stability and chemical stability.

**DUT-67** crystallizes in the *Fm*3̅*m* space group with a = 39.120 (5) Å, and the central metal zirconium ion is octa-coordinated by four carboxylate *O* atoms from TDC^2-^ ligands and the remaining four *O* atoms from four independent coordinated hydroxyl groups or water molecules and six zirconium atoms and eight TDC^2-^ ligands could form the nanocluster of [Zr_6_O_6_(OH)_2_ (tdc)_4_(CH_3_COO)_2_] (Supplementary Figure S3). In addition, **DUT-67** shows a binodal 8-connected three-dimensional framework with *reo* underlying net (Bon et al., 2013), which has the cuboctahedral cage with the diameter of 14.2 Å and the octahedral cage with the diameter of 11.7 Å (Figures 1C, D, and Supplementary Figure S4). The N_2_ adsorption and desorption curves of **DUT-67** at 77 K conform to type-I with a maximum adsorption of 261.6 cm^3^/g, and a calculated BET value of 1,031.9 m^2^/g (Bon et al., 2013) (Supplementary Figure S5).

### 2.2 Adsorption experiment

To reach the optimal conditions for the adsorption of the Cr(VI) ion from aqueous solution by **DUT-67**, four single-factor optimization experiments using the pH values of the Cr(VI) ion aqueous solution, the initial dose of **DUT-67**, the initial concentration of the Cr(VI) ion, and temperature were designed. In addition, the competition ion, material regeneration, kinetic, and thermodynamic experiments were discussed in detail.

**FIGURE 2:**
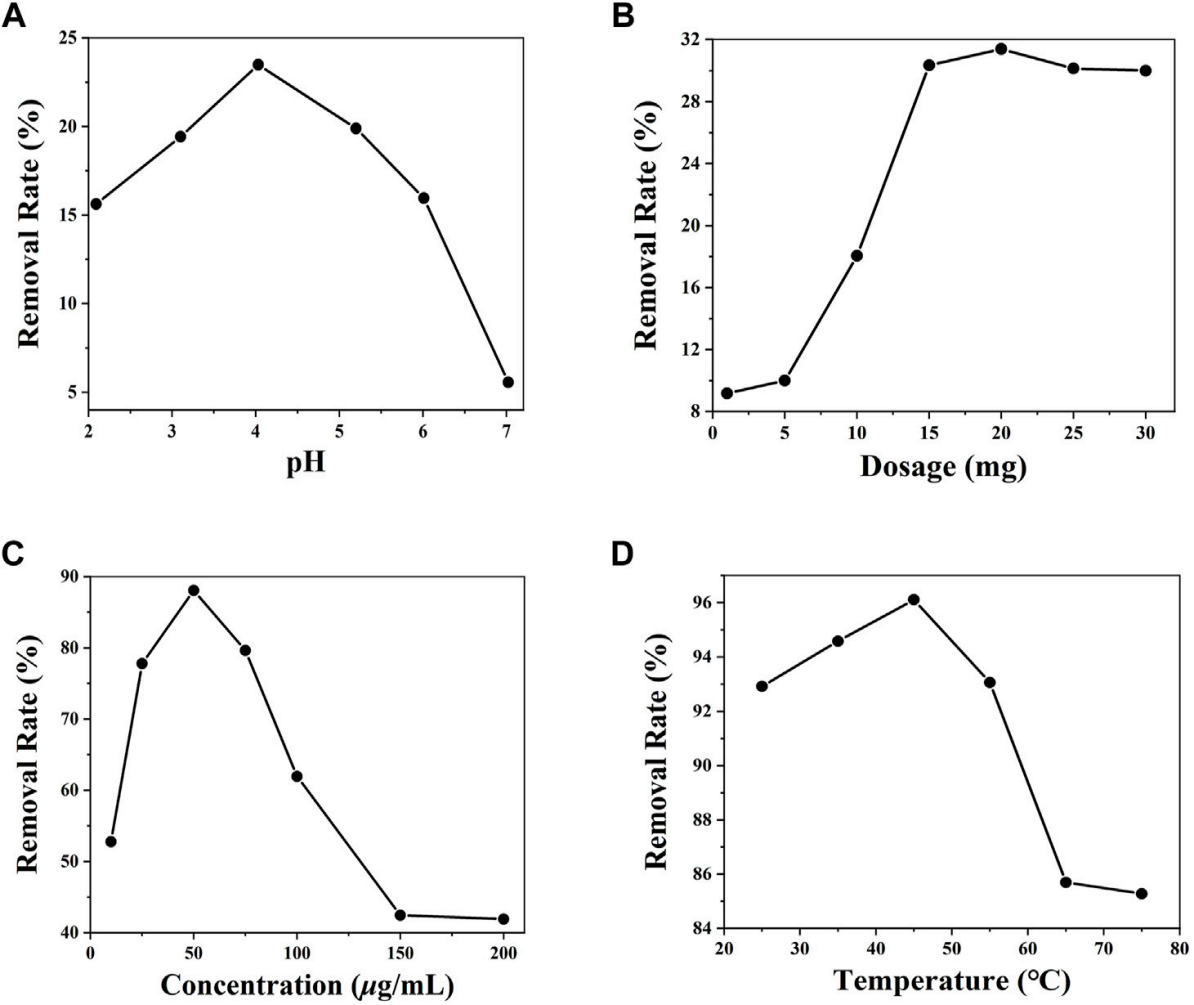
Influence of different pH **(A)**, dosages **(B)**, initial concentrations of the Cr(VI) ion, **(C)** and temperatures **(D)** on the removal by **DUT-67**.

The experimental results show that the removal rate gradually increases when the pH is between 2 and 4, while the removal rate decreases at pH between 5 and 7 (Figure 2A). When the pH value is between 2 and 5, the Cr(VI) ion is dominated by HCrO_4_^−^ and Cr_2_O_7_^2-^; when the pH value exceeds 7, the Cr(VI) ion is dominated by CrO_4_^2-^ (Li et al., 2021). Thus, the Cr(VI) ion aqueous solution with the highest removal rate was the solution with a pH value of 4.01. When the dose of **DUT-67** is less than 20 mg, the removal rate of the Cr(VI) ion increases, while the removal rate decreases when **DUT-67** dose exceeds 20 mg (Figure 2B). It is possible that the amount of adsorbent exceeds the maximum amount in a given volume; hence, its adsorption effect does not increase and the adsorption process is inhibited (Li et al., 2021). Therefore, the optimal dosage of **DUT-67** is 20 mg. When the initial concentration of the Cr(VI) ion exceeds 50 *μ*g/ml, the removal rate of **DUT-67** gradually decreases with the increase of Cr(VI) ion concentration. When the initial concentration of the Cr(VI) ion increases, the **DUT-67** removal rate reaches equilibrium and gradually decreases (Figure 2C). Thus, at the Cr(VI) ion concentration of 50 *μ*g/ml, the highest **DUT-67** removal rate of 88.06% is achieved. When the temperature ranges from 25°C to 45°C, the removal rate negligibly changes. However, when the temperature is higher than 45°C, the removal rate gradually decreases (Figure 2D). The most suitable removal temperature is 45°C with a removal rate of 96.2%. As the removal of the Cr(VI) ion by **DUT-67** is an endothermic reaction, the mass transfer rate of the Cr(VI) ion in the solution is accelerated in the process. In summary, the optimal removal conditions are as follows: the pH of the solution is 4.01, dose of **DUT-67** is 20 mg, initial concentration is 50 μg/ml, and adsorption temperature is 45°C, resulting in a maximum removal rate of 96.1%.

**FIGURE 3:**
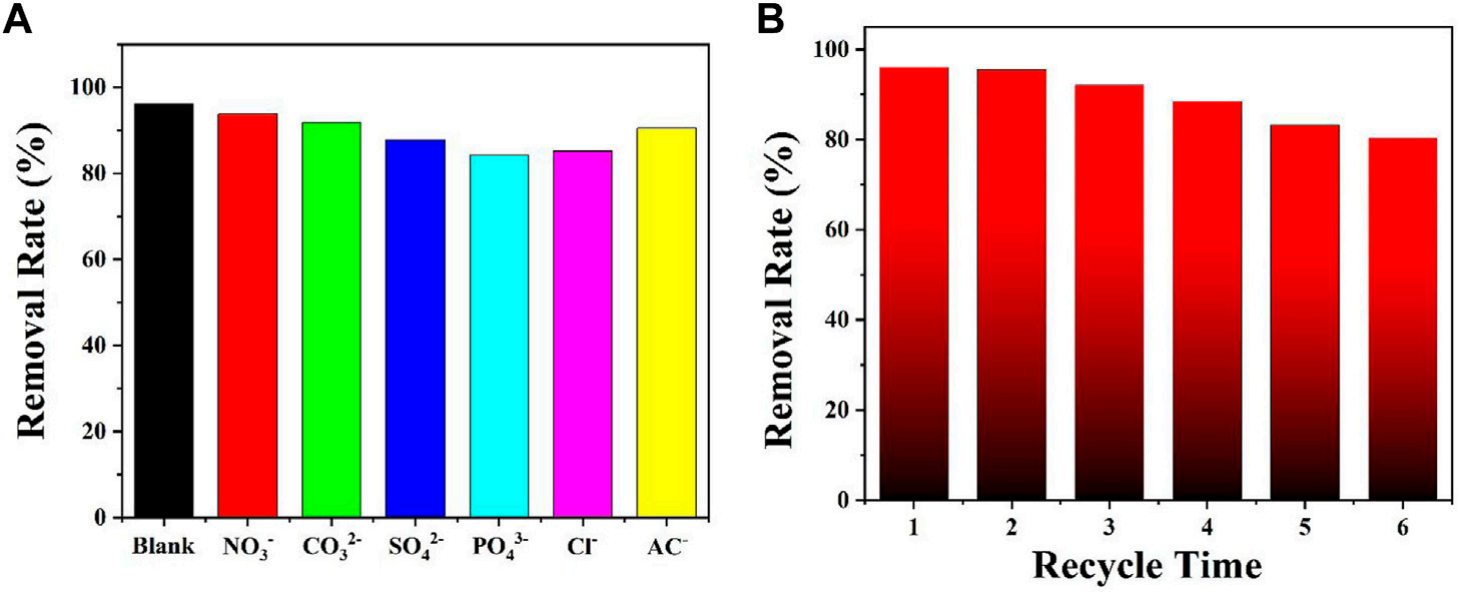
**(A)** Effect of co-existing ions on the removal of the Cr(VI) ion by **DUT-67**. **(B)** Recycling experiment on the removal of the Cr(VI) ion by **DUT-67**.

Regarding industrial wastewater containing a large number of co-existing anions such as NO_3_^−^, CO_3_^2-^, and SO_4_^2-^, investigation of the effect of co-existing ions on the removal of the Cr(VI) ion is essential (Shen et al., 2022). To study this effect, different co-existing anions were added into an aqueous solution of the Cr(VI) ion. The results indicated that the co-existing ions had only weak effects on the removal of the Cr(VI) ion (Figure 3A). Thus, **DUT-67** can maintain the removal rate for the Cr(VI) ion in the presence of co-existing ions. To study the recyclability of the material, the reusability of **DUT-67** after the adsorption of the Cr(VI) ion was further investigated (Zheng et al., 2019; Shen et al., 2022). After six recycles, the removal rate of 80.1% was observed, and the **DUT-67** framework maintained the crystal state (Figure 3B).

In addition, the adsorption equilibrium was gradually reached with the change of concentration and adsorption time, and the adsorption isotherm for the removal of the Cr(VI) ion by **DUT-67** with the maximum adsorption capacity is 105.42 mg g^−1^ (Supplementary Figure S6), whose BET and the maximum adsorption capacity exhibited a moderate level compared with other reported MOFs (Supplementary Table S1).

**FIGURE 4:**
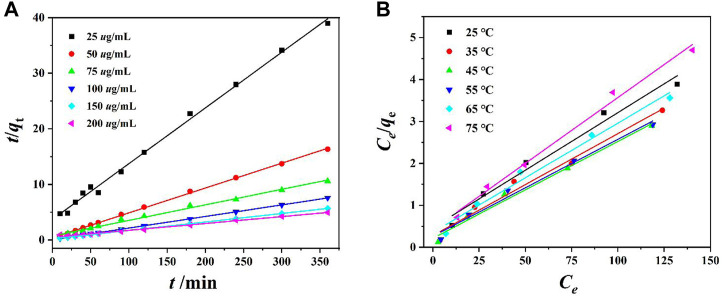
**(A)** Pseudo-second-order dynamic model fitting for Cr(VI) ion removal by **DUT-67**, and **(B)** Langmuir adsorption mode fitting for Cr(VI) ion removal by **DUT-67**.

Equations (3) and (4) (Supplementary Data Sheet 1) were used for fitting the pseudo-first-order kinetics and pseudo-second-order kinetics. The pseudo-first-order kinetics fitting curve is achieved by ln (*q*_e_-*q*_t_) and *t*, and the pseudo-second-order kinetics fitting curve is achieved by *t*/*q*_t_ and *t* (Yang et al., 2019; Shen et al., 2022). The experimental results show that the *R* ^2^ value of the pseudo-first-order kinetic model does not conform to the linear law, while the *R* ^2^ value of the pseudo-second-order kinetic model is very close to 1 (Figure 4A and Supplementary Figure S7). This indicates that the adsorption process of the Cr(VI) ion by **DUT-67** corresponds with the pseudo-second-order kinetic model with the rate-control process.

Equations (5) and (6) were used for fitting of the Langmuir and Freundlich models. *c*_e_ is plotted with *c*_e_/*q*_e_, to achieve the Langmuir isotherm, and the Freundlich isotherm is obtained by plotting ln*c*_e_ with ln*q*_e_ (Yang et al., 2019; Shen et al., 2022). The *R* ^2^ value of the Freundlich isotherm indicates a poor linear relationship, while the *R* ^2^ value of the Langmuir isotherm demonstrates higher linearity (Figure 4B and Supplementary Figure S8). This indicates the thermodynamic process of Cr(VI) ion removal by **DUT-67** conforms to the Langmuir model with the monolayer adsorption.

According to Equations (7) and 8), and 9), 1/T and ln*K* are regarded as the transverse coordinate and ordinate, respectively, and using them, the thermodynamic linear fitting curve is obtained (Wang et al., 2019; Shen et al., 2022). When the temperatures were 308, 318, and 328 K, the Gibbs free energy (△*G*) values were -0.135 kJ/mol, -0.524 kJ/mol, and -0.791 kJ/mol, respectively. The results indicated that the adsorption process of **DUT-67** on the Cr(VI) ion from an aqueous solution is a spontaneous and exothermic process adsorption process. In summary, according to the kinetic and thermodynamic experiment, the process of removal of Cr(VI) ions from an aqueous solution by **DUT-67** correlated with the pseudo-second-order kinetics model and Langmuir model, whose adsorption process belongs to weak chemical adsorption with a spontaneous and exothermic process (Babapour, et al., 2022; Valadi, et al., 2022; Wang, et al., 2022; Chen, et al., 2023; Yuan, et al., 2023).

**FIGURE 5:**
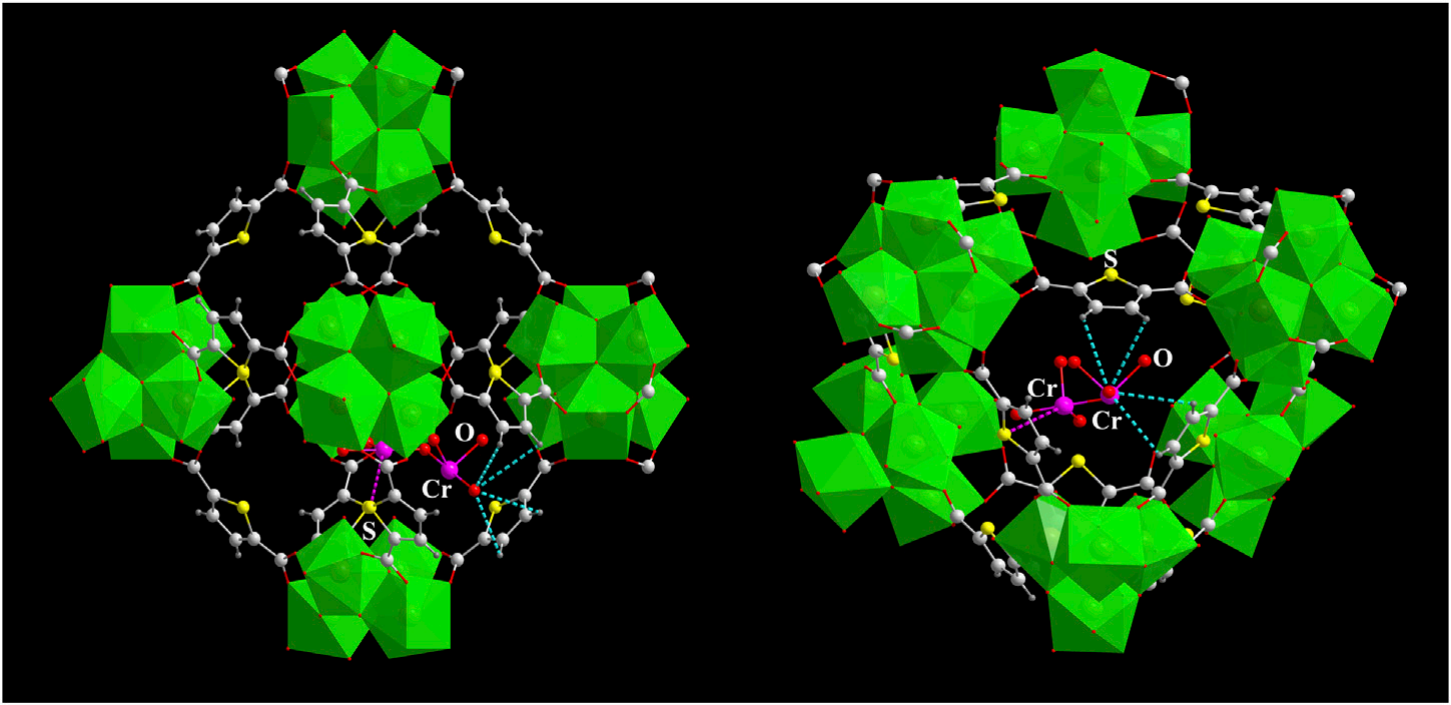
Interaction of the Cr(VI) ion and **DUT-67**.

To investigate the adsorption sites and the adsorption mechanism of the Cr(VI) ion in the pores of **DUT-67**, the adsorption locator module of Materials Studios 8.0 was used (Shen et al., 2022). The adsorption mechanism of the Cr(VI) ion by **DUT-67** might be *via* the hydrogen bonding interactions between the *O* atom from the dichromate and *H* atom from the TDC^2-^ ligand (Zheng et al., 2019; Li et al., 2021; Shen et al., 2022; Valadi, et al., 2022; Yuan, et al., 2023), the Cr(VI) atom from the dichromate, and the *S* atom from the TDC^2-^ ligand (Figures 5A,B), which play crucial roles in the adsorption of the Cr(VI) ion by **DUT-67**.

## 3 Conclusion

Robust **DUT-67** was prepared by the hydrothermal method and characterized by PXRD, TGA, and SEM. The effect of **DUT-67** on the adsorption process of the Cr(VI) ion from an aqueous solution under different conditions was studied, and the competing ion, material regeneration, kinetic, and thermodynamic experiments were explored. The results led to the determination of the optimal adsorption conditions yielding a maximum removal rate of 96.1% and a maximum adsorption capacity of 105.42 mg g^−1^ with selective adsorption and material regeneration. In addition, the process of removal of Cr(VI) ions from an aqueous solution by **DUT-67** correlated with the pseudo-second-order kinetics model and Langmuir model, and the adsorption mechanism of **DUT-67** was reasonably explained. Therefore, DUT-67 can be regarded as a multifunctional material that can effectively remove Cr(VI) ions from the wastewater. This study provides a promising method for the separation and removal of Cr(VI) ions from wastewater in the future.

## Data Availability

The original contributions presented in the study are included in the article/Supplementary Material; further inquiries can be directed to the corresponding author.
