# Supplementary material for: Robust DUT-67 material for highly efficient removal of the Cr(VI) ion from an aqueous solution
Source: Front Chem. 2023 Feb 28;11:1148073. doi: 10.3389/fchem.2023.1148073 (PMC10011714; doi:10.3389/fchem.2023.1148073)
Supplement: Supplementary file 1 [file DataSheet1.pdf]

Supplementary Material

Robust DUT-67 Material for Highly Efficient Removal of Cr(VI) Ion from Aqueous Solution

Yanqiong Shen[1], Qingsong Yang [1,2*], Yongqiang Gao [1,3], Jinjie Qian [4*], Qipeng Li [1*]

[1] College of Chemistry and Chemical Engineering, Zhaotong University, Zhaotong, 657000, P. R. China

[2] Shuifu No.1 Middle School, Zhaotong, 657000, P. R. China

[3] Kunming Real-E Foreign Language Middle School, Kunming, 650217, P. R. China

[4] College of Chemistry and Materials Engineering, Wenzhou University, Wenzhou, 325035, P. R. China

***Correspondence:** 812620583@qq.com, jinjieqian@wzu.edu.cn and qpli@ztu.edu.cn

MATERIALS AND METHODS

Materials and methods

All the chemical reagents were commercially purchased and used without further purification. The structure, thermal stability, and morphology of the **DUT-52** were characterized by using an X-ray powder diffraction (XRD), Thermogravimetric analyzer (TGA), and scanning electron microscopy (SEM). XRD-6000 X-ray powder diffractor (Shimadzu, Japan), FEI Quanta 200F scanning electron microscope (FEI), Thermogravimetric analyzer (Mettel-Toledo, Switzerland); ASAP 2020 (Mike Instruments); PHS-2F pH meter (Shanghai); UV-2802PCS spectrophotometer (Shanghai Unico Instruments Co., Ltd.).

Preparation of the DUT-67

$ZrCl_4$ (230 mg, 1 mol) were dissolved in 12.5 mL *N*, *N'*-dimethylformamide (DMF) and 12.5 mL *N*-Methyl pyrrolidone (NMP) in the 50 mL polytetrafluoroethylene reactor, which was sonicated for 10 min. The 2,5-thiophene dicarboxylic acid (115 mg, 0.67 mol) was added into the mixture solutions, which were sonicated for 5 min, and then 10 mL acetic acid were added and the mixture solutions was sonicated for 10 min, which were heated in an oven (120 ℃) for 48 hours and cooled to room temperature. The prepared samples were centrifuged, washed three times with fresh DMF, exchanged the solvent with acetone for 3 times, dried and activated under the vacuum for 4 h, obtained the white powder of the activated **DUT-67**.

Adsorption Experiment

A certain dosage of activated **DUT-67** were added into a certain amount of Cr(VI) ions aqueous solution with different concentration and different pH, which were oscillated at different temperatures and different times, the residual concentration of Cr(VI) ions were determined by the UV spectrophotometry.

$$R\% = \frac{c_0 - c_e}{c_0} \times 100\% \qquad (1)$$

$$q_e = \frac{(c_0 - c_e) \times V}{m} \qquad (2)$$

where c_0 is the initial concentration of the Cr(VI) ions (μg/mL), c_e is the equilibrium concentration of Cr(VI) ions (μg/mL), V is the volume of dichromate ions (mL), m is the dosage of adsorbent **DUT-67** (mg), q_e is the adsorption capacity (μg/mg).

The removal rate (R) and the adsorption capacity (q_e) of the **DUT-67** were calculated by Equations (1) and (2).

Competition Ion Experiment and Regeneration Experiment

20 mg **DUT-67** were added into 10 mL Cr(VI) ions aqueous solution (50 μg/mL) with different competition ions (NO_3^-, CO_3^{2-}, SO_4^{2-}, PO_4^{3-} and Cl^-) and pH (4.03) under 45 °C, which were oscillated for 30 min and the residual concentration of Cr(VI) ions was determined.

DUT-67 after adsorption of Cr(VI) ions (20 mg) was dispersed into the mixture (40 mL) of methanol and acetic acid (1:2, v/v). The resulting suspension was stirred for 12 h and the solid was collected through the concentration. Finally, the collected solid was washed with acetone and dried and activated under the vacuum for 4 h.

Adsorption Kinetics

20 mg **DUT-67** powder was added into 10 mL dichromate aqueous solution with different initial concentrations (25, 50, 75, 100, 150 and 200 μg/mL) and pH (4.03) under 45°C, which were oscillated different times and the residual concentration of Cr(VI) ions was determined.

$$\ln(q_e - q_t) = \ln q_e - k_1 t \qquad (3)$$

$$t/q_t = 1/k_2 q_e^2 + t/q_e \qquad (4)$$

where q_t is the adsorption capacity corresponding to t (μg/mg), q_e is the adsorption capacity (μg/mg), k_1 and k_2 are the kinetic rate constant and t is the adsorption time (m).

The kinetic relationship in the adsorption of Cr(VI) ions aqueous solution by the **DUT-67** were calculated based on the quasi-primary kinetic equation (3) and the quasi-secondary kinetic equation (4).

Adsorption Thermodynamics

20 mg **DUT-67** powder was added into 10 mL Cr(VI) ions aqueous solution with different initial concentrations (25, 50, 75, 100, 150 and 200 μg/mL) and pH (4.03) under different temperature (25,

35, 45, 55, 65 and 75 °C), which were oscillated for 30 min and the residual concentration of Cr(VI) ions was determined.

$$c_e/q_e = c_e/q_{max} + 1/q_{max}K_L \tag{5}$$

$$\ln q_e = \ln K_F + 1/n \ln c_e \tag{6}$$

where q_{max} is the maximum saturated adsorption capacity (μg/mg), q_e is the adsorption capacity (μg/mg), K_L is the Langmuir adsorption constant related to the adsorption energy, K_F is the Freundlich adsorption constant related to the adsorption capacity, n is a temperature-dependent constant and c_e is the equilibrium concentration of Cr(VI) ion (μg/mL).

The thermodynamic relationship in the adsorption of the Cr(VI) ion aqueous solution by the **DUT-67** were calculated based on the Langmuir equation (5) and the Freundlich equation (6).

$$K = q_e/c_e \tag{7}$$

$$\ln K = \triangle S/R - \triangle H/RT \tag{8}$$

$$\triangle G = -RT\ln K \tag{9}$$

Where K is the thermodynamic equilibrium constant, R is the gas adsorption constant (8.314 J/mol K), T is absolute temperature (K), $\triangle G$, $\triangle S$ and $\triangle H$ are the Gibbs free energy, Entropy and Enthalpy, respectively.

The $\triangle G$, $\triangle S$ and $\triangle H$ of the **DUT-67** can be calculated according to equations (7), (8) and (9).

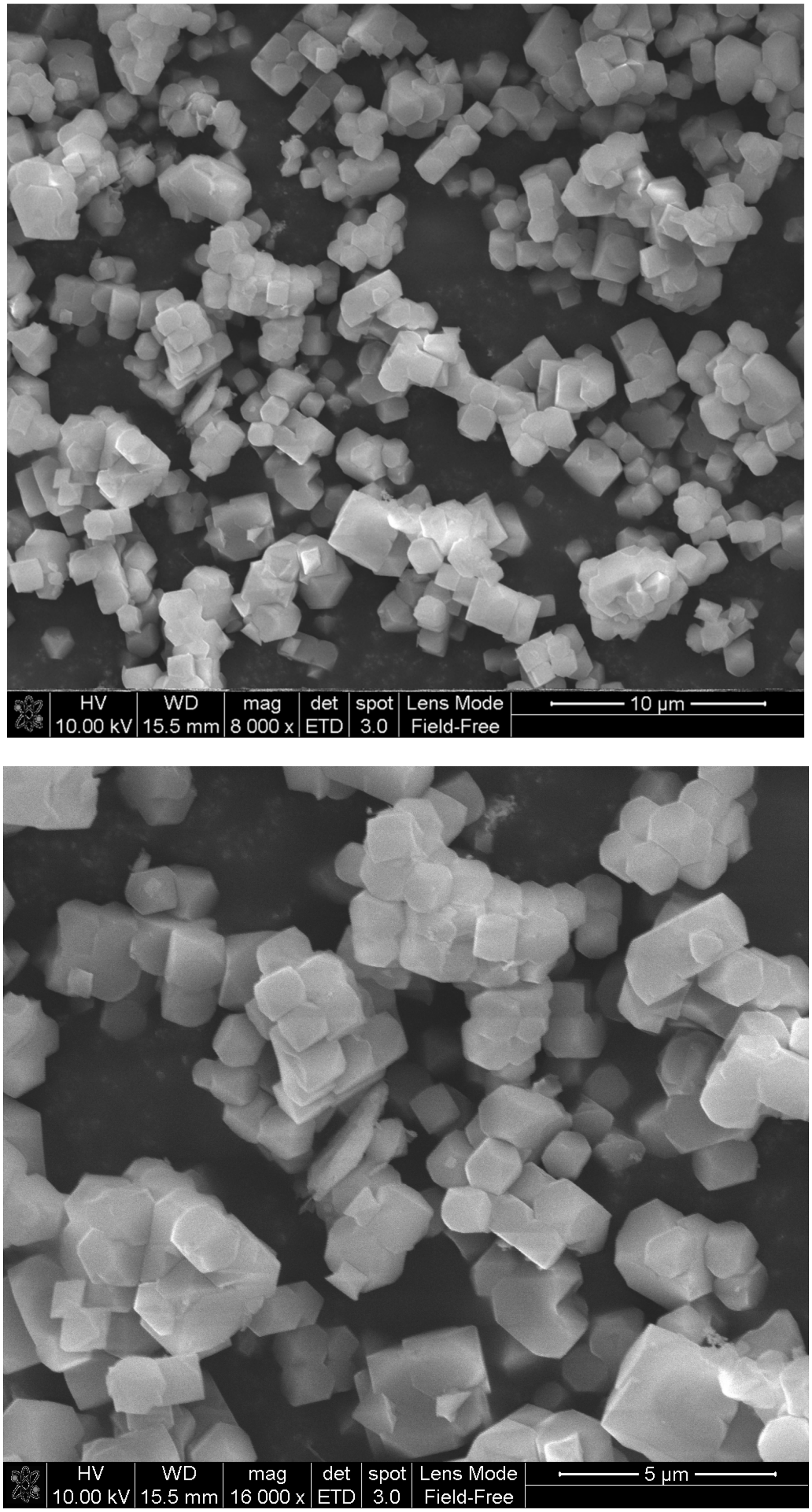

Figure S1. The SEM of **DUT-67**.

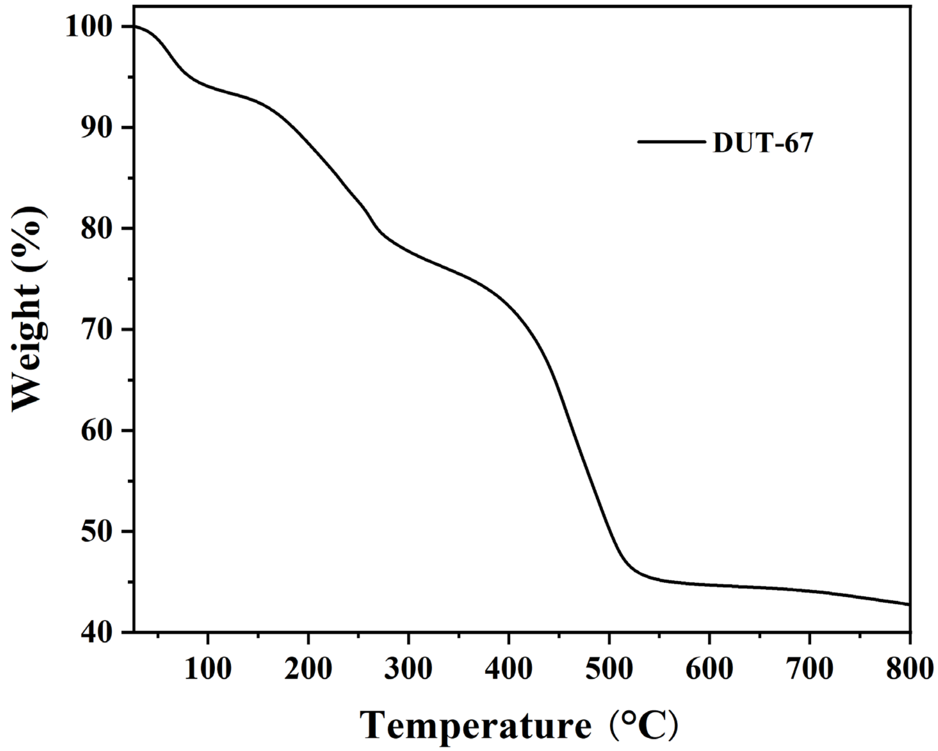

Figure S2. The TGA of **DUT-67**.

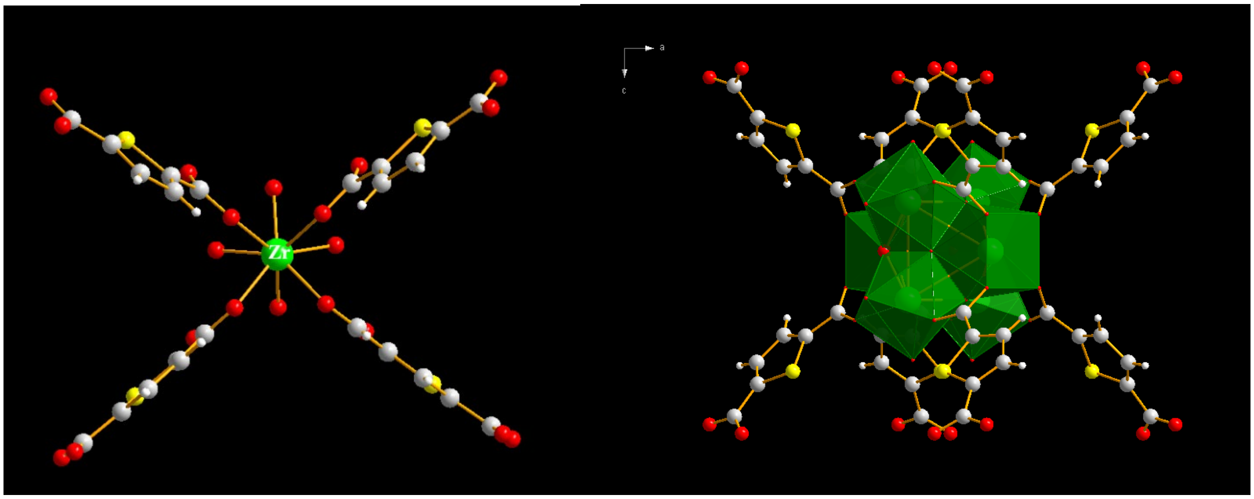

Figure S3. The coordination environment of the central metal zirconium ion and the SBUs structure in **DUT-67**.

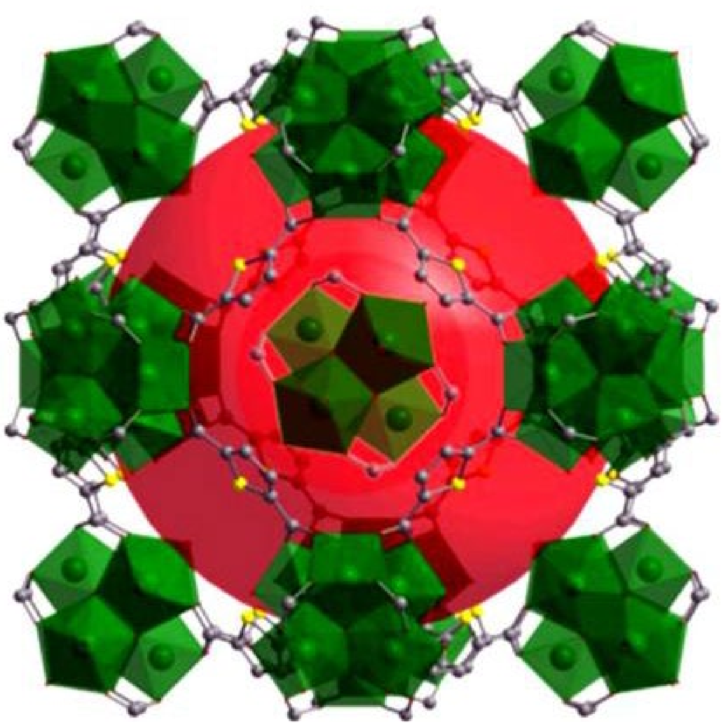

Figure S4. The nanocage of **DUT-67**.

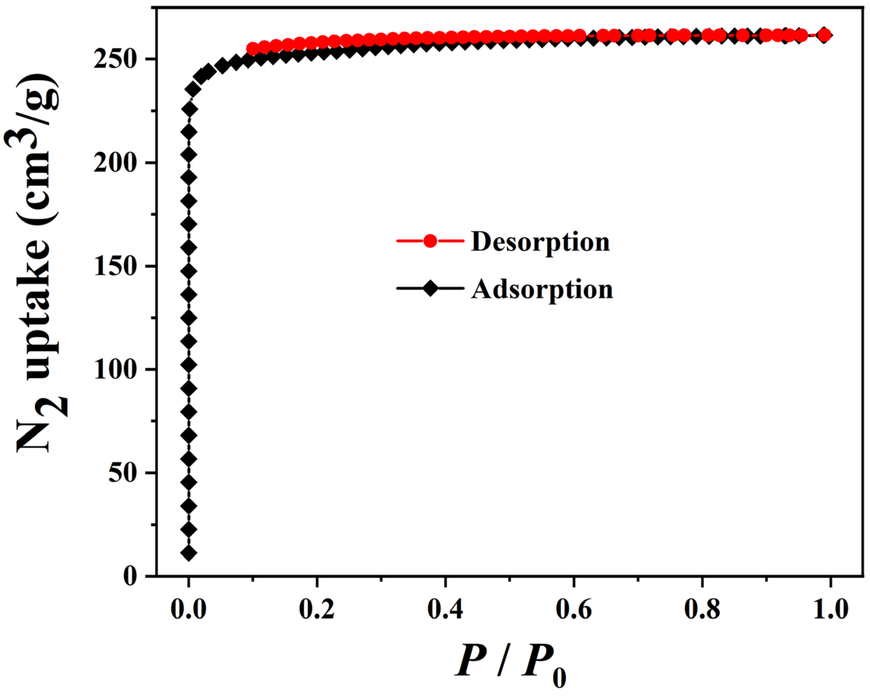

Figure S5. The N_2 isotherms of **DUT-67**.

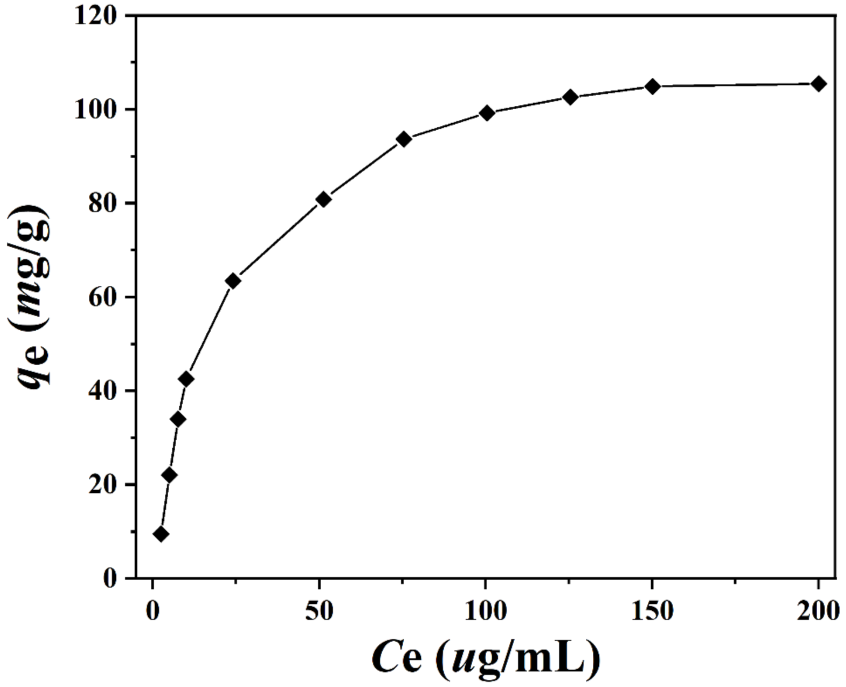

Figure S6. The adsorption isotherm for the removal of Cr(VI) ion by the **DUT-67.**

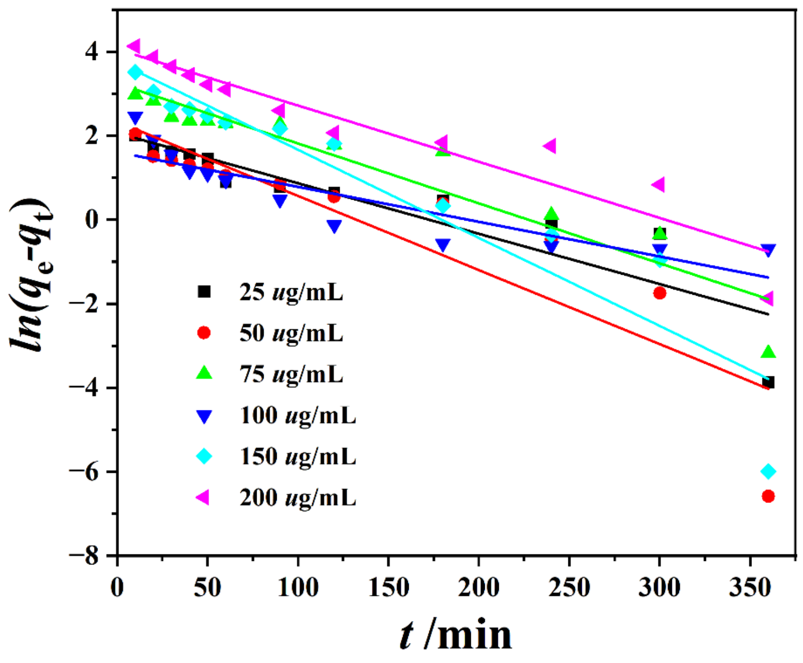

Figure S7. The pseudo first-order dynamic model fitting on the Cr(VI) ion by the **DUT-67**

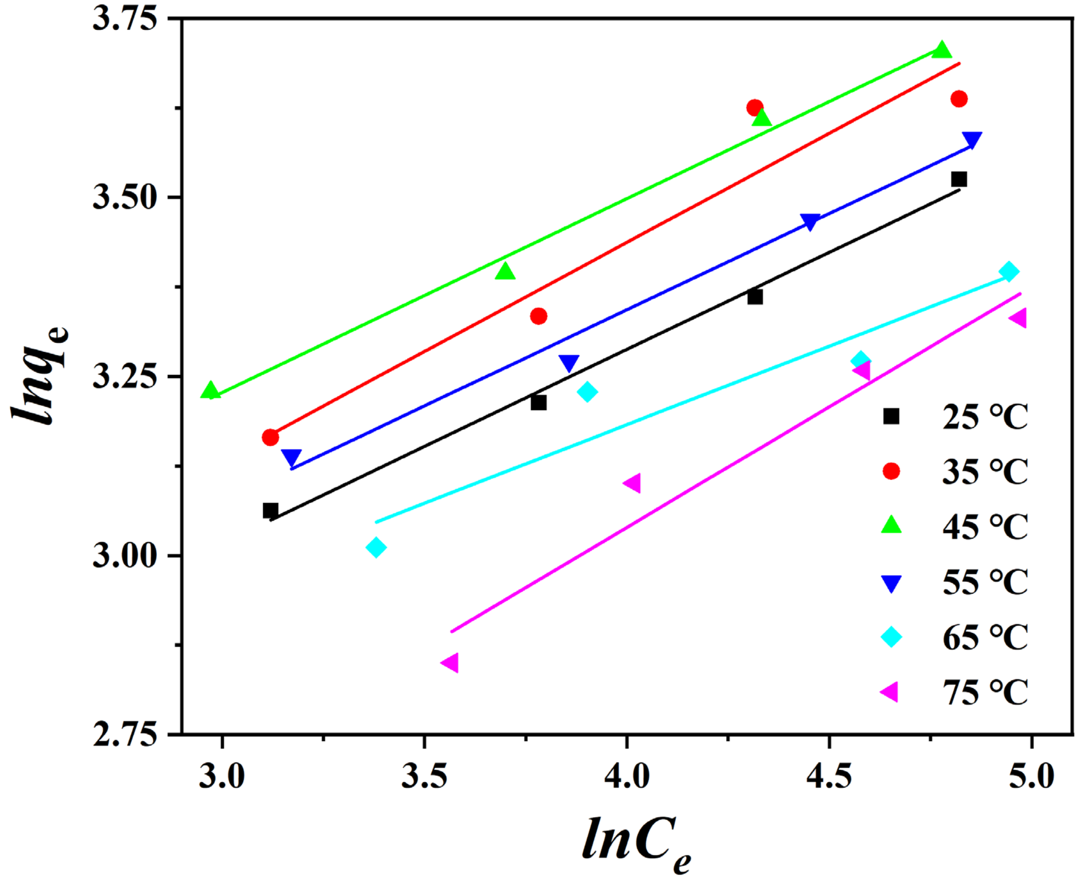

Figure S8. The Freundlich adsorption mode fitting for the removal of Cr(VI) ion by the **DUT-67**

Table S1 Cr(VI) ions adsorption performance of some reported MOFs

MOFs	Adsorbate	Adsorption quantity / (mg/g)	Ref
ABT-$(ClO_4)_2$	$Cr_2O_7^{2-}$	271	1-3
FIR-53	$Cr_2O_7^{2-}$	74.2	1-3
FIR-54	$Cr_2O_7^{2-}$	103	1-3
Cu-BTC	CrO_4^{2-}	48	1-3
$[Cu_4O(BDC)]_n$	Cr(VI)	43.9	1-3
ZJU-101	$Cr_2O_7^{2-}$	245	1-3
MONT-1	$Cr_2O_7^{2-}$	211.8	1-3
TMU-30	$HCrO_4^-$	145	1-3
NU-1000	$Cr_2O_7^{2-}$	76.8	1-3
UIO-66-NH_2	Cr(VI)	32.36	1-3
MIL-100(Fe)-Na_2CO_3	$HCrO_4^-$	46.02	1-3
ZIF-8@CA	Cr(VI)	41.8	1-3
BUT-39	$Cr_2O_7^{2-}$	215	1-3
TMU-66	$HCrO_4^-$	60.24	1-3
MOF-801	$HCrO_4^-$	156.2	1-3
Cu^{II}-MOF	$HCrO_4^-$	190	1-3
Zr-MSA	$HCrO_4^-$	202.0	1-3
Zr-DMSA	$HCrO_4^-$	138.7	1-3
NiCo-LDH	Cr(VI)	99.9	1-3
UPC-50	$Cr_2O_7^{2-}$	56.8	1-3
UIO-66	$Cr_2O_7^{2-}$	60.2	1-3
Dy-MOF	$Cr_2O_7^{2-}$	62.88	1-3
JLU-MOF60	$Cr_2O_7^{2-}$	149	1-3
DUT-52	$Cr_2O_7^{2-}$	120.68	1-3
**DUT-67**	$\mathbf{Cr_2O_7^{2-}}$	**105.42**	**This work**

Reference

(1) Li, Y. X.; Zhong, W. B.; Xie, L. H.; Xie, Y. B.; Li, J. R. Recent Advances in Adsorptive Removal of Cr(VI) Ions by Metal-Organic Frameworks. *Chinese J. Inorg. Chem.* **2021**, 37(3), 385-400.

(2) Zheng, M. Q.; Zhao, X. D.; Wang, K. K.; She, Y. B.; Gao, Z. Q. Highly Efficient Removal of Cr(VI) on a Stable Metal-Organic Framework Based on Enhanced H-Bond Interaction. *Ind. Eng. Chem. Res,* **2019**, 58, 23330-23337.

(3) Shen, Q., Duan, R., Qian, J., et al. (2022). Preparation of Highly Stable DUT-52 Materials and Adsorption of Dichromate Ions in Aqueous Solution. *ACS Omega.* 7(19), 16414-16421.
